# A New Positron Emission Tomography Probe for Orexin Receptors Neuroimaging

**DOI:** 10.3390/molecules25051018

**Published:** 2020-02-25

**Authors:** Ping Bai, Sha Bai, Michael S. Placzek, Xiaoxia Lu, Stephanie A. Fiedler, Brenda Ntaganda, Hsiao-Ying Wey, Changning Wang

**Affiliations:** 1 Chengdu Institute of Biology, Chinese Academy of Sciences, Chengdu 610041, China; pbai@mgh.harvard.edu (P.B.); luxx@cib.ac.cn (X.L.); 2 Athinoula A. Martinos Center for Biomedical Imaging, Department of Radiology, Massachusetts General Hospital, Harvard Medical School, Charlestown, MA 02129, USA; sbai@mgh.harvard.edu (S.B.); sfiedler@mgh.harvard.edu (S.A.F.); bNtaganda@mgh.harvard.edu (B.N.); hsiaoying.wey@mgh.harvard.edu (H.-Y.W.); 3 University of Chinese Academy of Sciences, Beijing 100049, China

**Keywords:** orexin receptors, PET, radiotracer, imaging

## Abstract

The orexin receptor (OX) is critically involved in motivation and sleep−wake regulation and holds promising therapeutic potential in various mood disorders. To further investigate the role of orexin receptors (OXRs) in the living human brain and to evaluate the treatment potential of orexin-targeting therapeutics, we herein report a novel PET probe ([^11^C]CW24) for OXRs in the brain. CW24 has moderate binding affinity for OXRs (IC_50_ = 0.253 μM and 1.406 μM for OX_1_R and OX_2_R, respectively) and shows good selectivity to OXRs over 40 other central nervous system (CNS) targets. [^11^C]CW24 has high brain uptake in rodents and nonhuman primates, suitable metabolic stability, and appropriate distribution and pharmacokinetics for brain positron emission tomography (PET) imaging. [^11^C]CW24 warrants further evaluation as a PET imaging probe of OXRs in the brain.

## 1. Introduction

Orexin (OX) is a hypothalamic hypocretin peptide that mediates multiple functions such as arousal, attention, neuroendocrine, water balance, and pain modulation [1,2,3,4,5,6]. The orexin system has two peptide members, orexin-A and orexin-B with 33 amino acids and 28 amino acids, respectively [7]. Orexin receptors (OXRs) also have two subtypes (OX_1_R and OX_2_R). Orexin-A has similar binding affinities to OX_1_R and OX_2_R, while orexin-B has a 10-fold higher affinity for OX_2_R compared with OX_1_R. OXRs are differently expressed throughout the brain. OX_1_R is mainly expressed in the prefrontal and infralimbic cortex, hippocampus, paraventricular thalamic nucleus, and locus coeruleus [8]. OX_2_R is mainly distributed in the cerebral cortex, septal nuclei, lateral hypothalamus, hippocampus, and hypothalamic nuclei [9]. The differential distribution of OXRs in the brain may be responsible for the various physiological and psychiatric functions mediated by the OXR system [10,11,12].

Therapeutics targeting the orexin system have been investigated for various brain disorders such as insomnia [1,3], cluster headache [13], substance abuse [4,14], and maladaptation to stress [15,16]. This has led to the design and evaluation of orexin antagonists, including dual-OX_1_R and OX_2_R antagonists (DORA) and selective OX_1_R and OX_2_R antagonists (SORA). [7] Almorexant [5] (ACT-426606) [17] from Actelion, SB-649868 [18] from GSK, and Suvorexant [6] from Merck are a few examples demonstrating the efforts from pharmaceutical companies. Among these compounds, many have entered into clinical trials, however, Suvorexant is the only U.S. Food and Drug Administration (FDA) approved orexin drug for the insomnia market. The discovery of ORX antagonists provided powerful tool compounds to investigate the roles of the orexin system in a variety of biological processes. However, the roles of the orexin system in the brain are not well understood and further investigations with translational methods, such as in vivo imaging, are warranted.

A non-invasive molecular imaging with positron emission tomography (PET) is a unique tool to study the living brain, which provides a distinct advantage to quantify receptor expression and/or drug occupancy of novel ligands in vivo. Over the last decade, though there have been major advances in the development of orexin antagonists [19,20,21,22,23,24,25], no PET radiotracer is available for OXR imaging in the human brain. This limits our ability to investigate the orexin system in the human brain and hinders the potential of orexin-targeting neurotherapeutics for brain disorders. 

**Figure 1:**
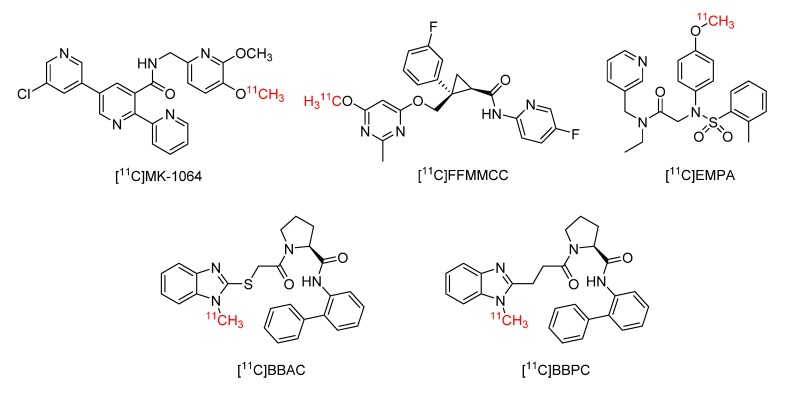
Structures of orexin receptor radioligands.

To bridge the gap, several orexin antagonists have been radiolabeled by our group, as well as others. Most of the early discovery attempts resulted in compounds with low brain uptake or strong P-glycoprotein (P-gp) binding, including [^11^C]MK-1064 [26], [^11^C]EMPA [27], [^11^C]BBAC [28], and [^11^C]CW4 [29] (Figure 1). Towards the development of a successful OXR brain-PET imaging probe, we report the discovery and evaluation of [^11^C]CW24 with good brain uptake, suitable metabolic stability, and pharmacokinetics based on animal imaging studies in rodents and non-human primates.

## 2. Results and Discussion

### 2.1. Selection of Scaffold for Orexin Imaging Probe Development

Several small molecules have been reported as DORA or SORA [20,30,31]. Previously, our group has radiolabeled several DORAs based on a 1,4-diazepane scaffold [27,29]. However, the low brain uptake limited their further evaluation as a brain-PET radiotracer. Recently, IPSU was reported as an OX_2_R selective antagonist with good brain uptake [32,33]. It has been studied in rodents, including blood and brain pharmacokinetics, as well as EEG (electrocorticogram/electroencephalogram) and EMG (electromyogram) recordings following treatment with these OXR antagonists in mice. Based on the structure-activity relationship (SAR) study of ISPU analogs, methylation of the indole imine does not disrupt OXR antagonism [32]. Hence, we chose the indole imine of IPSU as the radiolabeling site and synthesized CW24. The affinity of CW24 for orexin receptors (OX_1_R and OX_2_R) in cells was measured in a competition binding assay against [^125^I] orexin. CW24 displayed potent activity at OXRs (IC_50_ 0.253 µM OX_1_R, 1.406 µM OX_2_R). Compared to IPSU binding, this was ~5 fold (OX_1_R) and ~2 fold (OX_2_R) increase in potency, warranting further evaluation.

### 2.2. Physicochemical Properties of CW24

**Figure 2:**
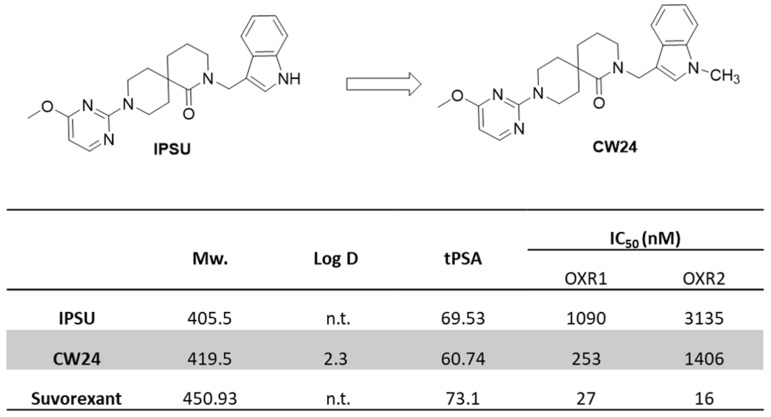
Structures and physicochemical properties of IPSU and CW24 (n.t. = not tested). IC50 of IPSU, CW24 and suvorexant were measured by a radioligand competition binding assay.

Lipophilicity (logP) and molecular weight need to be optimized for sufficient blood-brain barrier (BBB) penetration while also avoiding high non-specific binding. Studies have shown that it is preferred for clogP ≤ 4, clogD ≤ 3, total polar surface area (tPSA) between 30–75 and molecular weight (M.W.) < 500 for successful brain-PET imaging probes [34,35]. IPSU is reported as a non-selective OXR antagonist with good brain uptake [33]. CW24, the methylated form of IPSU, had favorable properties for CNS penetration (M.W. = 419.5, Log D_(7.4)_ = 2.3, tPSA = 60.74, IC_50_ = 0.253 μM and 1.406 μM for OX_1_R and OX_2_R, respectively) (Figure 2). Furthermore, we tested the off-target binding of CW24 in vitro, [36] and were pleased to see that CW24 was selective for OXRs compared to 40 other CNS targets(National Institute of Mental Health Psychoactive Drug Screening Program, NIMH PDSP) (Table S1).

### 2.3. Chemical Synthesis for CW24 and [^11^C]CW24

**Scheme 1:**
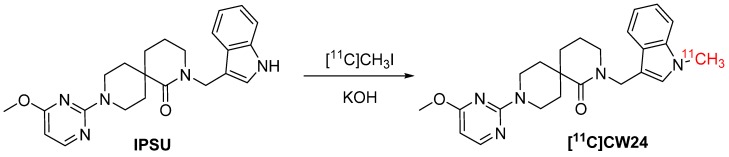
Radiolabeling condition: IPSU (precursor, 0.5 mg), [^11^C]CH_3_I, KOH (10 mg), in 1.0 mL DMF, 3 min, 100 °C. Radiochemical yield (RCY): 10–21% (non-decay corrected from trapped [^11^C]CH_3_I).

CW24 was prepared from IPSU via direct methylation in the presence of a base (Figures S1 and S2). [^11^C]CW24 was radiosynthesized from IPSU (0.5 mg) in DMF (1 mL) with KOH (10 mg) and allowed to react with [^11^C]CH_3_I for 3 min at 100 °C (Scheme 1). The average synthesis time was 30–35 minutes from end of cyclotron bombardment (EOB) to end of synthesis (EOS), the radiochemical yield (RCY) was 10–21% (non-decay corrected, from trapped [^11^C]CH_3_I), the specific activity (A_s_) was 1.28 ± 0.2 mCi/nmol (EOS), the chemical and radiochemical purities of [^11^C]CW24 were ≥ 97%.

### 2.4. Mouse Imaging with [^11^C]CW24

**Figure 3:**
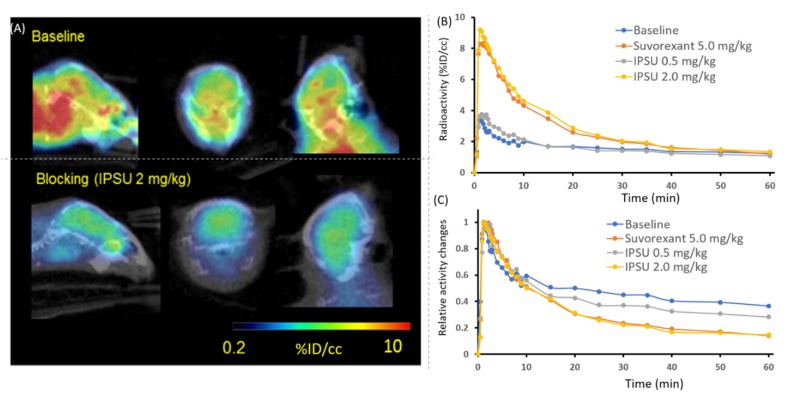
(**A**) The PET/CT imaging of [^11^C]CW24 focused on mice brain (20‒60 min after intravenous administration (i.v.)); (**B**) baseline and blocking time-activity curve (TAC) of [^11^C]CW24 and (**C**) TAC after normalized the brain uptake curves with the highest uptake time point (20 seconds post-injection).

We performed PET-computed tomography (CT) imaging on mice to test [^11^C]CW24 as a neuroimaging probe for OXR imaging in vivo. [^11^C]CW24 exhibited high brain uptake (%ID/cc = 3.5% at C_max_, 1.5 min post-injection). In general, %ID/cc above 0.1% in rat or 0.01% in non-human primate within 5 min post-injection is above the threshold for suitable BBB penetration for CNS PET imaging studies [37]. Next, to evaluate the specificity of [^11^C]CW24 for OXRs, PET-CT imaging studies were performed in mice after 5 min pretreatment with IPSU (0.5 and 2.0 mg/kg; i.v.) or Suvorexant (5.0 mg/kg; i.v.). Time-activity curves (TACs) were normalized for peak uptake (~20 seconds post-injection in these studies) (Figure 3). Approximately 10% reduction in [^11^C]CW24 uptake with 0.5 mg/kg IPSU administration in 30–60 mins post-injection of [^11^C]CW24. In addition, approximately 50% reduction was found when the blocking dose of IPSU was increased to 2.0 mg/kg. Suvorxant (5.0 mg/kg) administration at 30–60 mins post-injection of [^11^C]CW24 also showed ~50% reduction in the mouse brain. Following IPSU pretreatment, [^11^C]CW24 binding was reduced in a dose-dependent manner in the brain for the relative radioactivity changes after administration. These results strongly support that [^11^C]CW24 binds selectively to the OXRs in vivo.

### 2.5. Non-human Primate (NHP) Imaging with [^11^C]CW24

**Figure 4:**
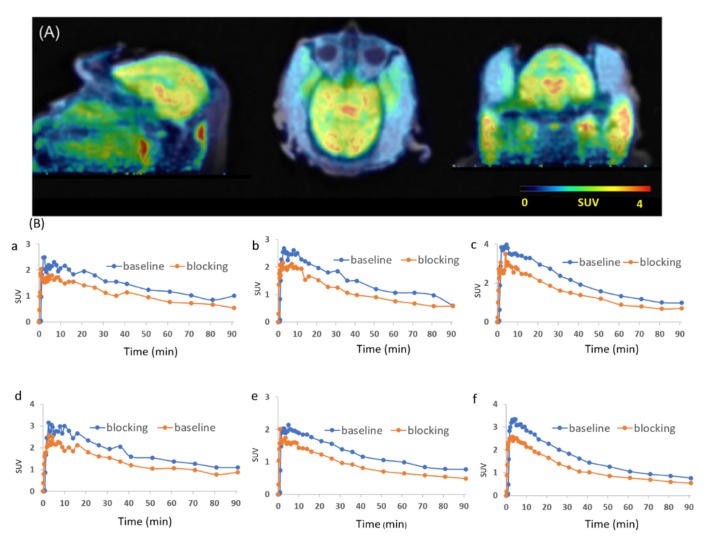
(**A**) Positron emission tomography (PET)-Magnetic resonance (MR) Imaging (macaque brain). Summed PET images (30–90 min after [^11^C]CW24 injection) superimposed structural MRI from the same macaque. (**B**) Time−activity curves for brain regions of interest from baseline and blocking scans are shown (2.0 mg/kg IPSU) (a) hippocampus, (b) nucleus accumbens, (c) thalamus, (d) hypothalamus, (e) putamen and (f) cerebellum.

**Figure 5:**
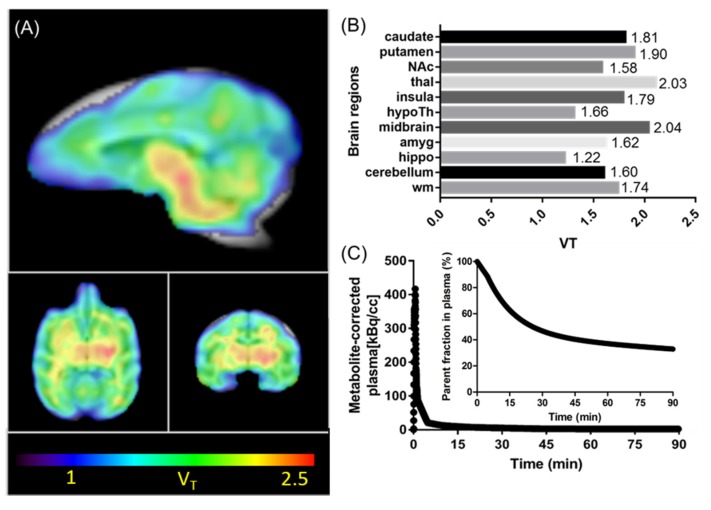
Kinetic modeling results with [^11^C]CW24 in the macaque brain. (**A**) The total volume of distribution (V_T_) images derived from Logan plot with arterial blood data as input function (color bar indicates V_T_ values from 1–2.5 mL/cm^3^ (blue to red); (**B**) Regional V_T_ values showed that the expression difference in brain regions; (**C**) Arterial plasma analysis showed that [^11^C]CW24 radioactivity was rapidly cleared from blood and [^11^C]CW24 stability evaluated in plasma over time showed lasting presence of ~47% of parent compound at 30 min.

Encouraged by the promising imaging results of [^11^C]CW24 in mice, we performed PET- Magnetic resonance imaging (MR) imaging in a NHP (Figure 4 and Figure 5). In macaque, [^11^C]CW24 demonstrated high brain uptake. We observed a peak standard uptake value (SUV) (SUV=C(T)/(injected dose/body weight) between 1.5–3.0 for all brain regions examined (Figure 4A). Furthermore, relatively higher uptake was observed in the midbrain and the thalamus and lower uptake in the hippocampus which suggests heterogenous expressions of OXR in the macaque brain. To further investigate the specific binding of [^11^C]CW24, a blocking study was performed in which 2.0 mg/kg IPSU was administered 5 min prior the PET-MR acquisition. Similar to the blocking results shown in mice, a reduction of radioactivity uptake was found in several brain regions including hippocampus, nucleus accumbens, thalamus, hypothalamus, putamen and cerebellum (Figure 4B). Using the arterial blood data as input function, Logan graphical analysis was applied to quantify volume of distribution (V_T_, mL/cm^3^) of [^11^C]CW24 (Figure 5). The metabolite-corrected arterial plasma after [^11^C]CW24 bolus injection is showed in Figure 5C.

Based on our evaluation both in rodents and NHPs in vivo, [^11^C]CW24 is a promising OXR PET radioligand candidate. It has been reported that OXR was typically present in the cerebral cortex, septal nuclei, hippocampus, medial thalamic groups, raphe nuclei, and abundantly expressed in hypothalamic nuclei that regulate the homeostasis in rodents [12]. In addition, OX_2_R was found to have expression in the hypothalamus that enables its unique role of sleep-wake regulation [11]. Compare to the previous OXR probes we developed [27,29] [^11^C]CW24 had higher brain uptake, increased isoform selectivity over IPSU, appropriate kinetics and distribution, which warrants further development of OXR PET radioligands based on [^11^C]CW24.

## 3. Materials and Methods

### 3.1. Synthesis of Compound CW24

A mixture of IPSU (5 mg, 0.012 mmol, purchased form MedChemExpress), KOH (4 mg, 0.06 mmol), and CH_3_I (5 mg, 0.036 mmol) in DMF (0.1 mL) were reacted at room temperature overnight. The reaction mixture was purified by reverse phase chromatography (H_2_O: CH_3_CN = 1:1) to give the desired product (2 mg, 0.005 mmol, 39% yield) as a white solid. ^1^H-NMR (500 MHz, Chloroform-d) δ 8.04 (d, *J* = 5.6 Hz, 1H), 7.64 (d, *J* = 7.9 Hz, 1H), 7.29 (d, *J* = 8.2 Hz, 1H), 7.22 (t, *J* = 7.7 Hz, 1H), 7.10 (t, *J* = 7.4 Hz, 1H), 7.02 (s, 1H), 5.93 (s, 1H), 4.72 (s, 2H), 4.40 (dt, *J* = 13.7, 4.7 Hz, 2H), 3.87 (s, 3H), 3.75 (s, 3H), 3.38 (m, 2H), 3.24 (t, *J* = 6.1 Hz, 2H), 2.24 (m, 2H), 1.83–1.77 (m, 2H), 1.73 (m, 2H), 1.50 (m, 2H).^13^C-NMR (125 MHz, Chloroform-d) δ 174.50, 169.94, 161.78, 157.98, 137.12, 128.79, 127.61, 121.89, 119.68, 119.41, 110.84, 109.29, 95.96, 53.00, 46.79, 41.77, 40.27, 39.80(2), 33.84(2), 32.82, 30.61, 18.91. LC-MS [M]^+^ 419.85. 

### 3.2. Radiosynthesis of [^11^C]CW24

[^11^C]CH_3_I was trapped in a reactor (TRACERlab FX-M synthesizer, General Electric) preloaded with the precursor (0.5 mg), KOH (5.0 mg) in 1.0 mL dry DMF. The mixture was stirred for 3 min at 100 °C and followed by adding water (1.2 mL). The product was separated by reverse phase semi-preparative HPLC (Phenomenex Luna 5u C8(2), 250 mm × 10 mm, 5 μm; 5.0 mL/min; 60% H_2_O + ammonium formate (0.1 M)/ 40% CH_3_CN; isocratic). The collected final product was loaded onto a C-18 sep-pak cartridge, and rinsed with water (5 mL), eluted with EtOH (0.3 mL), and saline (0.9%, 2.7 mL). The average time required for the synthesis from EOB to EOS was 30–35 min. The average radiochemical yield (RCY) was 10%–21% (non-decay corrected to trapped [^11^C]CH_3_I). Chemical and radiochemical purities were ≥ 95 % (measured with HPLC equipped with a UV detector and a gamma detector) with a specific activity (A_s_) of 1.28 ± 0.2 mCi/nmol (EOS).

### 3.3. Assessment of Lipophilicity (Log D; pH 7.4)

Log D was determined according to methods identical to those we previously reported [38].

### 3.4. Human Orexin GPCR Binding (Agonist Radioligand) Assay

Radioligand competition binding assays were performed by Panlabs, Eurofins Pharma Discovery Services. Briefly, human recombinant orexin OX1 receptors expressed in CHO-S cells were used in modified HEPES buffer pH 7.4. An aliquot was incubated with 0.1 nM [^125^I] Orexin A for 60 minutes at 25 °C. Receptors were filtered and then counted to determine [^125^I] Orexin A specifically binding. Human recombinant OX2 receptors were used in modified HEPES buffer pH 7.4. An aliquot was incubated with 0.04 nM [^125^I] Orexin A for 180 minutes at 25 °C. Membranes were filtered and then counted to determine [^125^I] Orexin A specifically bound.

### 3.5. Rodent PET/CT Acquisition

The Subcommittee on Research Animal Care (SRAC) serves as the Institutional Animal Care and Use Committee (IACUC) for the Massachusetts General Hospital (MGH). SRAC reviewed and approved all procedures detailed in this paper. B6C3F1/J mice (male, 18-month old; *n* = 4 total) were utilized in this study. IPSU and Suvorexant were purchased form MedChemExpress and dissolved in 1.0 % DMSO + 1.0 % Tween 80 + 98.0 % saline to make a solution of 1.0 mg/mL.

Animals were anesthetized with 1–1.5% isoflurane and maintained with isoflurane during the imaging scan. In a single imaging session, mice were arranged in a Triumph PET/CT scanner (Gamma Medica, Northridge, CA). The mice were administered [^11^C]CW24 (3700–7400 KBq per animal) after 5-min pre-treatment of IPSU (0.5 and 2.0 mg/kg; *n* = 1 for each dose; i.v.), Suvorexant (5.0 mg/kg; *n* = 1; i.v.), or vehicle (*n* = 1) via a lateral tail vein catheter. Animals underwent a 60 min dynamic PET scan followed by computed tomography (CT).

### 3.6. Rodent PET/CT Image Analysis

PET data were reconstructed using a 3D-MLEM method resulting in a full width at half-maximum resolution of 1 mm. PET and CT images in DICOM format were imported to PMOD (PMOD Technologies, Ltd. Zürich, Switzerland) and co-registered to the brain atlas. Volumes of interest (VOIs) were drawn as spheres in brain regions guided by CT structural images and summed PET data. Time-activity curves (TACs) were exported as activity per unit volume (%ID/cc) for analysis.

### 3.7. Macaque PET-MR Acquisition

A male rhesus macaque (14.4 kg) was included in this study. After endotracheal intubation, the macaque was catheterized antecubitally for radiotracer injection and a radial arterial line was placed for arterial blood sampling and radiometabolite analysis. The animal was anesthetized with 1.2–1.3% Isoflurane in medical oxygen throughout the imaging session. PET-MR data was acquired on a 3T Siemens MRI scanner (Munich, Germany) with a BrainPET insert. A baseline and a blocking scan were performed on the same animal. Dynamic PET image acquisition was initiated followed by a bolus administration of [^11^C]CW24 (179 MBq for the baseline scan and 192 MBq for the blocking scan). To characterize the specific binding of [^11^C]CW24, a blocking study was carried out in which 2.0 mg/kg IPSU (1.0 % DMSO + 1.0 % Tween 80 + 98.0 % saline to make a solution of 1.0 mg/mL) was administered 5 min prior the acquisition. A T1-weighting multi-echo magnetization-prepared rapid gradient-echo (MEMPRAGE) sequence began as the PET scan for anatomic co-registration. Dynamic PET data were corrected for decay, scatter, and attenuation and reconstructed using methods similar to our previous studies [39].

### 3.8. PET-MR Imaging Data Analysis for the Macaque Study

Motion-corrected PET data were registered to the INIA19 macaque MRI Template. VOIs were selected according to the INIA19 Template and NeuroMaps Atlas [40]. TACs from the whole *Macaque* brain as well as a few brain regions were exported. With the availability of a metabolite-corrected arterial plasma data, the regional volume of distribution (V_T_) was calculated using Logan Plot analysis (with a fixed t* of 40 min).

### 3.9. Plasma and Metabolite Analysis

Briefly, arterial blood samples collected (every 10 s at first 3 min post-administration of [^11^C]CW24, then 5 min, 10 min, 20 min, 30 min, 45 min, 60 min, and 90 min) during macaque imaging were centrifuged to separate plasma, which was then removed and placed in an automated gamma counter [41]. Metabolite analysis of blood samples collected from 5 to 90 min was conducted on a custom automated robot fitted with Phenomenex SPE Strata-X 500 mg solid phase extraction cartridges that were primed with ethanol (2 mL) and deionized water (20 mL). Protein precipitation was achieved by the addition of plasma (300 μL) to acetonitrile (300 μL), which was centrifuged for 1 min to obtain protein-free plasma (PFP). Three hundred microliters of PFP/acetonitrile solution was diluted into deionized water (3 mL), loaded onto the C18 cartridge, and removed of polar metabolites with 100% water. Next, a series of extractions was performed using water and acetonitrile. Each sample was counted in a WIZARD2 Automatic Gamma Counter to determine the presence of radiolabeled metabolites.

## 4. Conclusions

In summary, our developed PET imaging radio-ligands, [^11^C]CW24, provides a non-invasive quantitative imaging tool for evaluating OXR expression in the brain. Based on the in vitro and in vivo evaluations, [^11^C]CW24 had high brain uptake and good target-selectivity, but suffers from high non-specific binding. Therefore, [^11^C]CW24 could be used as a lead compound to develop brain-PET probes for OXRs to investigate not only the roles of OXR in a variety of disease applications, but also the development of OXR neurotherapeutics. In future studies, it is important that we characterize and optimize [^11^C]CW24 to improve specific binding for developing potential PET imaging probes for human imaging.

## Supplementary Materials

The following are available online, Figure S1: Representative 1H, 13C-NMR and HPLC data for CW24; Figure S2: The HPLC profile of CW24; Table S1: Off-target binding data of CW24.
