# Supplementary material for: A New Positron Emission Tomography Probe for Orexin Receptors Neuroimaging
Source: Molecules. 2020 Feb 25;25(5):1018. doi: 10.3390/molecules25051018 (PMC7179119; doi:10.3390/molecules25051018)
Supplement: Supplementary file 1 [file molecules-25-01018-s001.pdf]

A New Positron Emission Tomography Probe for Orexin Receptors Neuroimaging

Ping Bai[1,2,3], Sha Bai[2], Michael S. Placzek[2], Stephanie A. Fiedler[2], Brenda Ntaganda[2], Hsiao-Ying Wey[2,*], Changning Wang[2,*]

[1]Chengdu Institute of Biology, Chinese Academy of Sciences, Chengdu 610041, PR China

[2]Athinoula A. Martinos Center for Biomedical Imaging, Department of Radiology, Massachusetts General Hospital, Harvard Medical School, Charlestown, MA 02129, USA

[3]University of Chinese Academy of Sciences, Beijing 100049, PR China

*To whom correspondence should be addressed:

Changning Wang, PhD

Martinos Center for Biomedical Imaging at Massachusetts General Hospital, Harvard Medical School

149 13th Street, Suite 2301

Charlestown, MA 02129

cwang15@mgh.harvard.edu

Hsiao-Ying Wey, PhD

Martinos Center for Biomedical Imaging at Massachusetts General Hospital, Harvard Medical School

149 13th Street, Suite 2301

Charlestown, MA 02129

HsiaoYing.Wey@mgh.harvard.edu

Fig. S1. Representative ^{1}H, ^{13}C NMR and HPLC data for CW24.

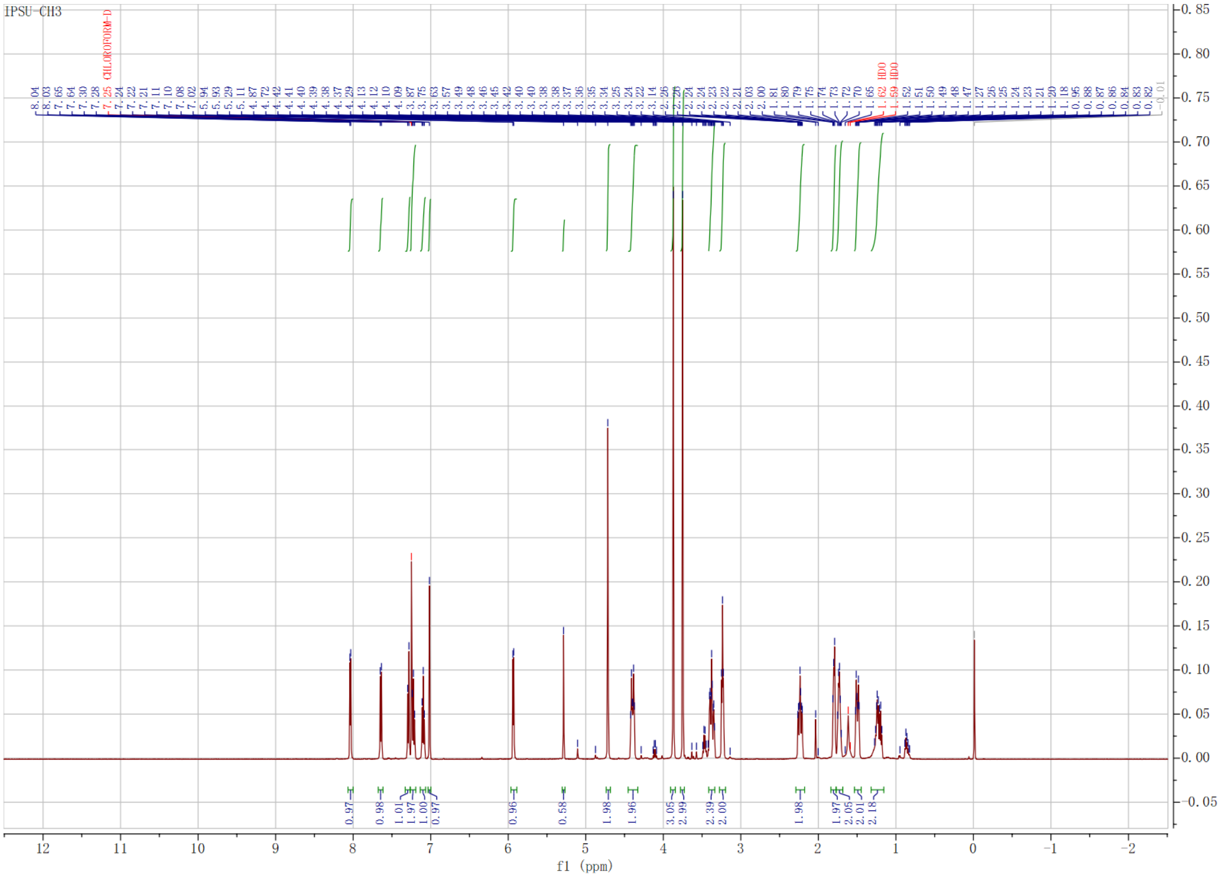
IPSU-CH3
f1 (ppm)

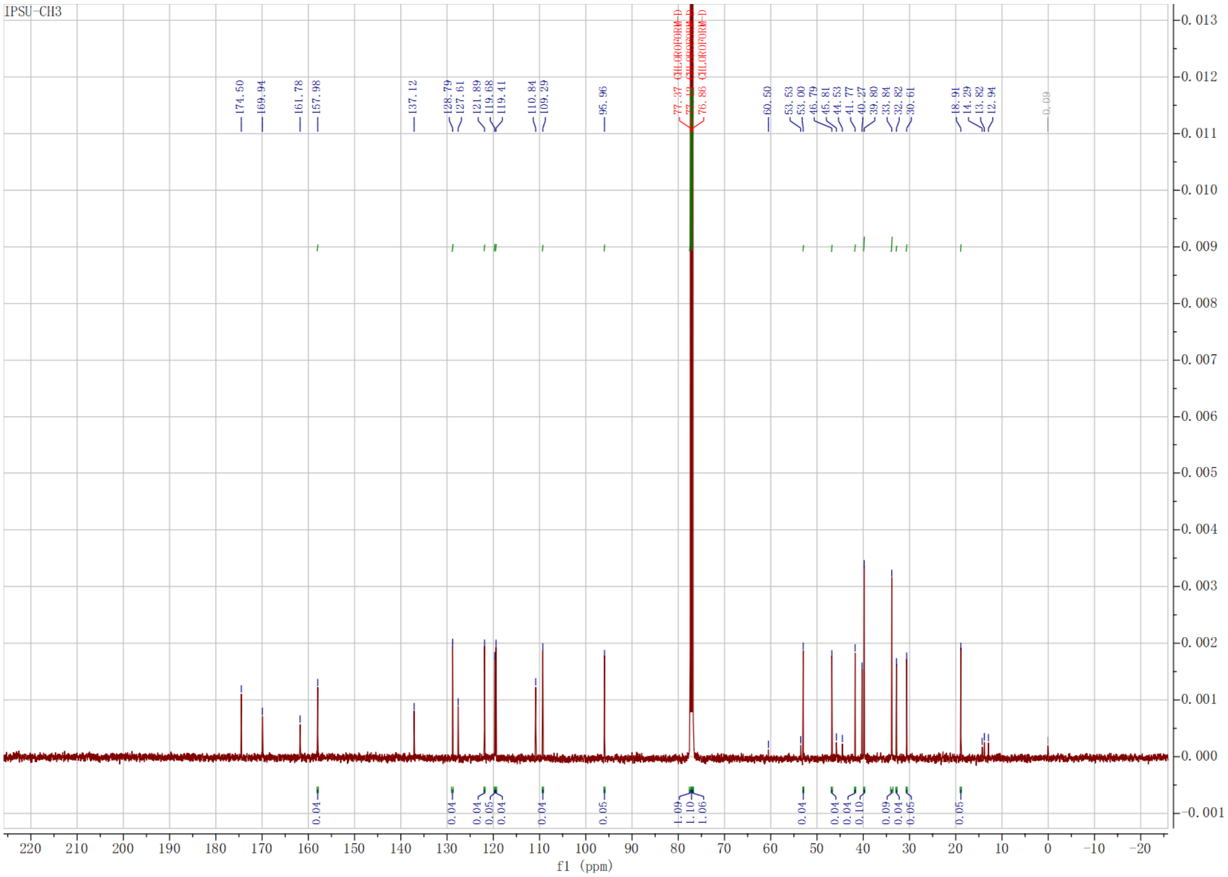

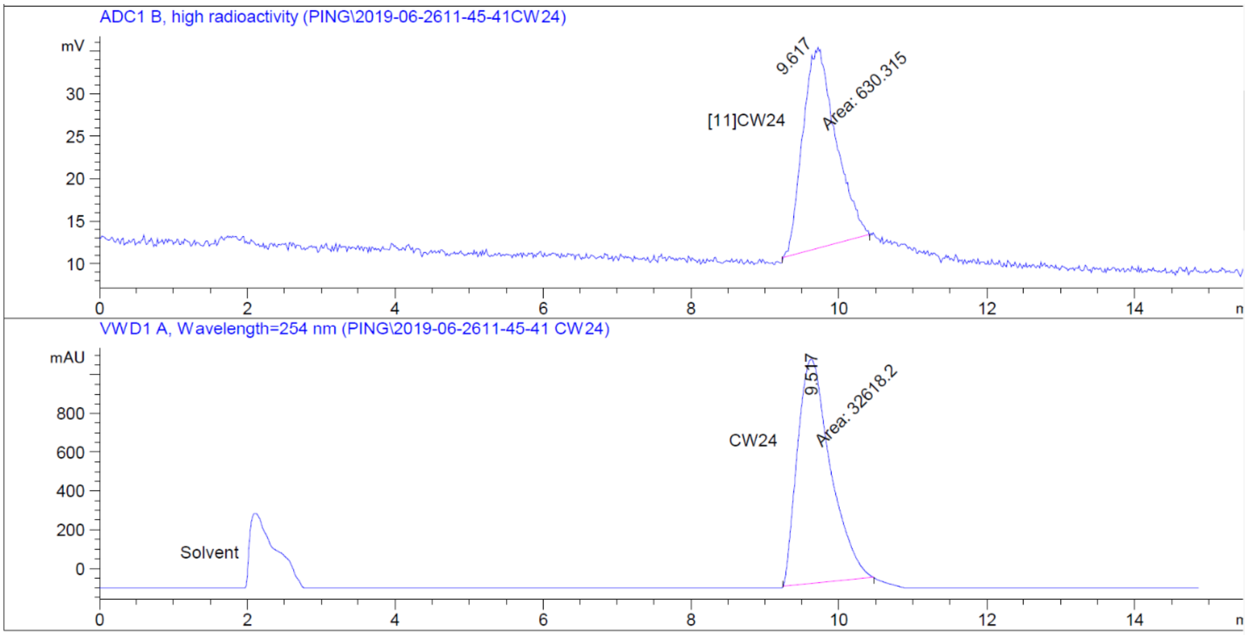

Fig. S2. The HPLC profile of CW24 (HPLC conditions: 60% H_2O + 0.1M AF/40% CH_3CN, at the flow rate of 5.0 mL/min.)

Table S1. Off binding data of CW24.

The off-target binding assays were performed at PDSP with their standard protocol (https://pdspdb.unc.edu/pdspWeb/content/UNC-CH%20Protocol%20Book.pdf). In radioligand binding assays, CW24 was tested at a single concentration (10 μM) and in quadruplicate in 96-well plates. The binding assays were carried out in a final of volume of 125 μl per well in appropriate binding buffers. The hot ligand concentration is usually at a concentration close to the Kd (unless otherwise indicated). Total binding and nonspecific binding are determined in the absence and presence of 10 μM of the appropriate reference compound, respectively. In brief, plates are usually incubated at room temperature and in the dark for 90 mins. Reactions are stopped by vacuum filtration onto 0.3% polyethyleneimine (PEI) soaked 96-well filter mats using a 96-well Filtermate harvester, followed by three washes with cold wash buffers (for details, see Tables 2 to 24). Scintillation cocktail is then melted onto the microwave-dried filters on a hot plate and radioactivity is counted in a Microbeta counter.

Receptor	% Inhibition 1	% Inhibition 2	% Inhibition 3	% Inhibition 4	Mean±SD % inhibition
5-HT1A	-4.55	-4.08	-22.12	-9.84	-10.15 ± 8.4
5-HT1B	18.96	17.99	6.23	10.41	13.4 ± 6.1
5-HT1D	32.06	19.98	11.86	30.24	23.54 ± 9.4
5-HT1E	31.33	19.5	22.46	2.07	18.84 ± 12.2
5-HT2A	25.7	20.15	12.75	-9.45	12.29 ± 15.4
5-HT2B	45.92	11.35	11.15	17.78	21.55 ± 7.7
5-HT2C	28.83	-38.5	8.5	58.2	14.26 ± 40.6
5-HT3	-7.18	2.23	-23.93	12.89	-4 ± 15.6
5-HT5A	5.59	-6.33	-13.11	-3.89	-4.44 ± 7.73
5-HT6	17.7	23.24	-12.28	-5.73	5.73 ± 17.3

5-HT7A	-92.69	-50.78	-54.31	-21.23	-54.75 ± 29.3
Alpha1A	17	-16.24	-17.64	-15.07	-7.99 ± 16.7
Alpha1B	-6.11	-16.27	-36.74	-23.55	-20.67 ± 12.8
Alpha1D	6.91	-32.75	-9.43	-19.42	-13.67 ± 16.7
Alpha2B	-8.15	-23.65	-14.5	8.29	-9.5 ± 13.4
Alpha2C	38.57	2.3	-9.91	13.27	11.06 ± 20.6
Beta2	-12.23	-5.09	-7.41	-17.26	-10.5 ± 5.4
D1	22.73	-0.84	21.85	28.02	17.94 ± 12.8
D2	-8.12	21.42	-24.23	4.13	-1.7 ± 19.3
D3	3.09	-9.41	-6.79	-6.5	-4.9 ± 5.5
D4	73.21	69.99	60.34	65.33	67.22 ± 5.6
D5	4.57	-5.32	7.05	55.75	15.51 ± 27.3
DAT	-0.27	-4.72	8.47	14.9	4.6 ± 8.8
DOR	41.02	8.06	10.63	-3.98	13.93 ± 19.1
GABAA	24.87	0.04	4	17.31	11.56 ± 11.5
H1	13	-0.52	1.94	27.5	10.48 ± 12.7
H2	25.4	24.43	24.78	22.12	24.18 ± 1.43
H3	-25	-12.5	0	0	-9.38 ± 11.92
H4	-8.25	9.91	8.32	3.76	3.44 ± 8.21
M1	17.45	-8.79	-6.38	-1.13	0.29 ± 11.93
M2	-1.12	-4.61	-17.35	22.75	-0.08 ± 16.71
M3	-5.69	-3.65	-12.4	-16.48	-9.56 ± 5.94
M4	0.88	-23.89	-3.92	11.5	-3.86 ± 14.83
M5	15.35	31.89	40.55	21.65	27.36 ± 11.13
MOR	5.06	-11.35	8.74	-10.61	-2.04 ± 10.43

SERT	10.62	-3.59	10.62	25.65	10.83 ± 11.94
Sigma 1	8.39	1.56	8.99	30.38	12.33 ± 12.50
Sigma 2	42.39	19.45	26.56	20.46	27.22 ±10.59
